# Studies on the mechanism of the lactide polymerization with highly active zinc guanidine catalysts

**DOI:** 10.1186/1758-2946-3-S1-P22

**Published:** 2011-04-19

**Authors:** I dos Santos Vieira, J Börner, U Flörke, S Herres-Pawlis

**Affiliations:** 1 Anorganische Chemie II, Technical University of Dortmund, 44225 Dortmund, Germany; 2 University of Paderborn, 33098 Paderborn, Germany

## 

Polylactide (PLA) is a biodegradable polyester which is able to replace petrochemical based plastics in many fields [1]. It is commonly produced by ring-opening polymerisation (ROP) of the cyclic diester lactide with metal containing initiator systems using anionic ligand systems [2].

Zinc complexes with neutral guanidine ligands have proven to be highly active initiators in the ROP of lactide [3,4]. In an integrated study combining kinetic analyses, spectroscopic measurements and DFT calculations the mechanism of the lactide polymerisation with this special catalyst class could be clarified. We could show that the polymerisation proceeds via a coordination-insertion-mechanism.

A complete reaction coordinate diagram including enthalpies of intermediates and transition states could be compiled by B3LYP-DFT for the initiation step of the polymerisation with the initiator [Zn(TMGqu)_2_(CF_3_SO_3_)][CF_3_SO_3_] and also for the chain propagation. The initiating step includes three transition states with 102 kJ/mol as highest activation barrier. The model for the propagation step reveals two transition states with an energy barrier not exceeding 65 kJ/mol. The results of this study demonstrate that lactide ROP proceeds not only with classical complexes using anionic ligands, but also with complexes containing neutral but highly nucleophilic guanidine ligands.
